# Exploring the mechanism of resistance to sorafenib in two hepatocellular carcinoma cell lines

**DOI:** 10.18632/aging.104195

**Published:** 2020-11-21

**Authors:** Zhi Zhang, Cheng-Zu He, Ya-Qin Qin, Jian-Jun Liao, Shang-Tao Huang, Steven Mo, Hong-Mian Li, Jian-Yan Lin

**Affiliations:** 1 Department of Hepatobiliary Surgery, The Fifth Affiliated Hospital of Guangxi Medical University, Nanning 530022, Guangxi, China; 2 Department of Oncology, the People’s Hospital of Binyang County, Binyang 530405, Guangxi, China; 3 Department of Liver Disease, The Affiliated Nanning Infectious Disease Hospital of Guangxi Medical University and The Fourth People’s Hospital of Nanning, Nanning 530023, Guangxi, China; 4 YuanDong International Academy of Life Sciences, Nanning 530229, Guangxi, China; 5 Department of Medical Laboratory Center, The Fifth Affiliated Hospital of Guangxi Medical University, Nanning 530022, Guangxi, China; 6 Department of Public Health, The Affiliated Nanning Infectious Disease Hospital of Guangxi Medical University and The Fourth People’s Hospital of Nanning, Nanning 530023, Guangxi, China

**Keywords:** sorafenib, hepatocellular carcinoma, drug resistance, miRNA, transcription factors

## Abstract

Sorafenib has long been the only approved systemic therapy for advanced hepatocellular carcinoma (HCC), but most patients show primary or acquired drug resistance. In the present study, RNA was extracted from sorafenib-resistant and -sensitive clones of the HCC cell lines HepG2 and Huh7. Protein-protein interaction networks of the up- and down-regulated genes common to the two sorafenib-resistant cell lines were extracted and subjected to modular analysis in order to identify functional modules. Functional enrichment analysis showed the modules were involved in different biological processes and pathways. These results indicate that sorafenib resistance in HCC is complicated and heterogeneous. The potential regulators of each functional module, including transcription factors, microRNAs and long non-coding RNAs, were explored to construct a comprehensive transcriptional regulatory network related to sorafenib resistance in HCC. Our results provide new insights into sorafenib resistance of HCC at the level of transcriptional regulation.

## INTRODUCTION

Hepatocellular carcinoma (HCC) is one of the world's deadliest cancers [1]. HCC can be diagnosed in early stages by ultrasonography, when it may still be treated by liver resection or transplantation, resulting in a 5-year survival rate above 50%. [2] In fact, several other therapies can treat early-stage HCC, including radiofrequency ablation, radioembolization and transarterial chemo embolization [3]. However, 70% of patients are diagnosed with HCC when the disease is already in a late stage. Only one systemic therapy is available for advanced HCC: sorafenib [4], which inhibits the proliferation of tumor cells by inhibiting Raf kinase and several receptor tyrosine kinases [5]. Unfortunately, sorafenib provides benefit to only 30% of patients with advanced HCC, in part because many patients develop resistance to the drug within 6 months [4].

The mechanism of sorafenib resistance (SR) in HCC is poorly understood. Some studies have identified individual molecules that may confer SR, such as PHGDH [6], the Akt pathway activator SNHG1 [7] and PMPCB [8]. This led us to hypothesize that SR is caused by multiple molecular modules. To explore this possibility, we used two HCC cell lines to identify genes differentially expressed in the presence of SR, based on which we developed a comprehensive transcription regulatory network.

## RESULTS

**Figure 1:**
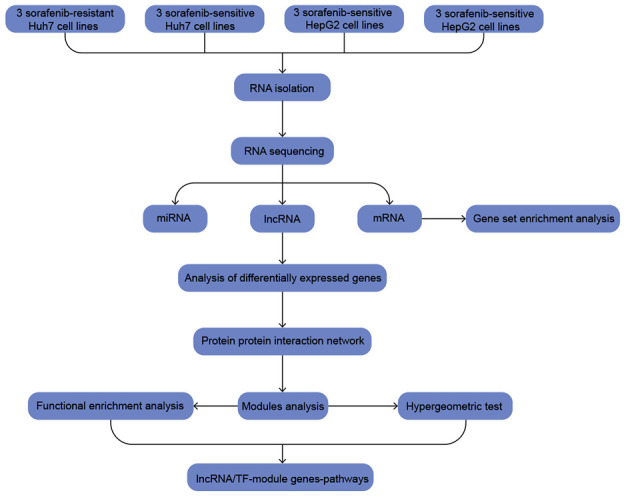
**The workflow of the present study.**

Using HepG2 and Huh7 cell lines, we compared SR and sorafenib-sensitive (SS) cells in order to identify Differentially expressed genes (DEGs) associated with SR. The DEGs common to both cell lines were then used to identify functional modules and regulatory molecules that may help identify molecular pathways and networks associated with SR (Figure 1).

### SR is associated with multiple genes and pathways in the two HCC cell lines

In the HepG2 cell line, 3075 genes were up-regulated and 638 down-regulated in association with SR. The corresponding numbers in Huh7 cells were 6946 and 2310 (Figure 2A). The sorafenib-targeted genes up-regulated in association with SR were ABCB1, ABCC4, ABCG2, BRAF and RALBP1 in the HepG2 line (Figure 2B); and ABCC2, ABCG2, CYP2B6, CYP2C19, CYP3A7, RAF1, RALBP1, and UGT1A1 in the Huh7 line (Figure 2C). Cluster analysis showed clear separation of DEGs between SR and SS cells of both lines (Figure 2D, 2E). We extracted 1630 up-regulated DEGs common to the two cell lines (Figure 2F) and 132 down-regulated genes common to the two lines (Figure 2G). Gene set enrichment analysis identified enrichment of the following pathways in SR HepG2 cells: apoptosis, the MAPK signaling pathway, and the p53 signaling pathway. Pathways enriched in SR Huh7 cells were the p53 signaling pathway, pathways in cancer, and the VEGF signaling pathway (Figure 2H).

**Figure 2:**
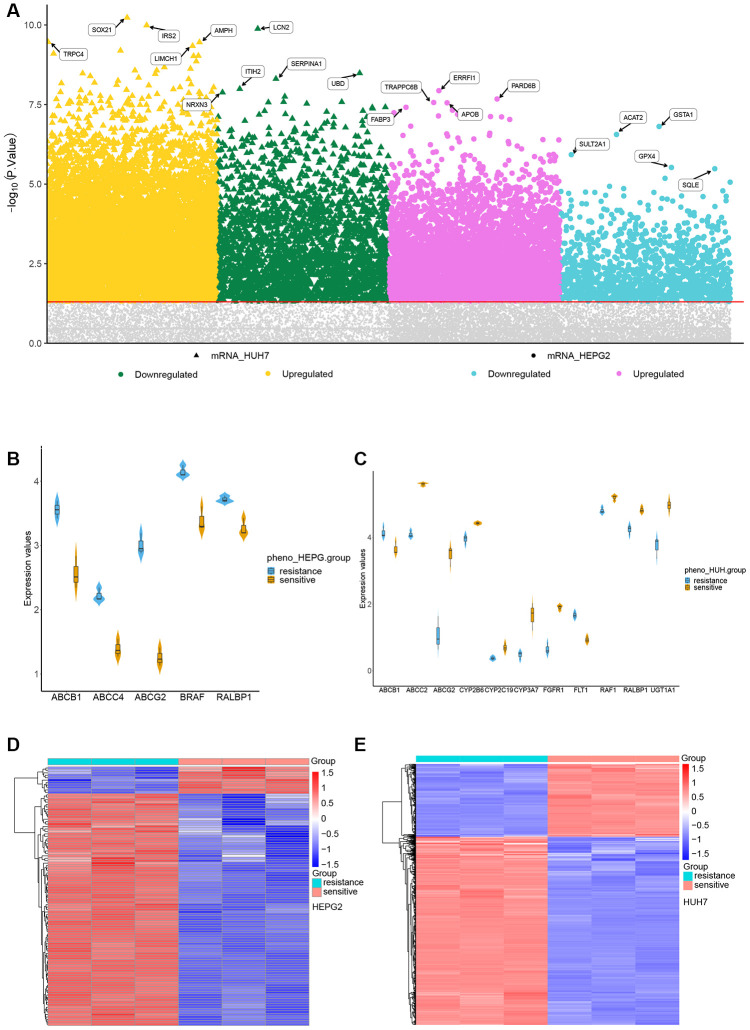
**Identification of differentially expressed genes (DEGs) in sorafenib resistance.** (**A**) DEGs in sorafenib resistance. (**B**) Expression of sorafenib-targeted genes in sorafenib-sensitive and -resistant HepG2 cells. (**C**) Expression of sorafenib-targeted genes in sorafenib-sensitive and -resistant Huh7 cells. (**D**) Cluster heatmap of DEGs between sorafenib-sensitive and -resistant HepG2 cells. (**E**) Cluster heatmap between sorafenib-sensitive and -resistant Huh7 cells.

**Figure 2:**
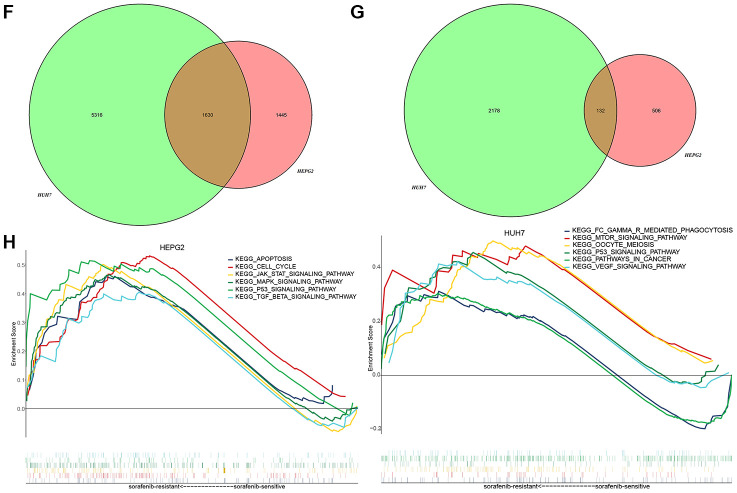
**Identification of differentially expressed genes (DEGs) in sorafenib resistance.** (**F**) Genes up-regulated in association with sorafenib resistance in HepG2 and Huh7 cells. (**G**) Genes down-regulated in association with sorafenib resistance in HepG2 and Huh7 cells. (**H**) Gene set enrichment analysis of genes up- or down-regulated in association with sorafenib resistance in both HCC cell lines.

### SR is associated with perturbations in multiple functional modules

**Figure 3:**
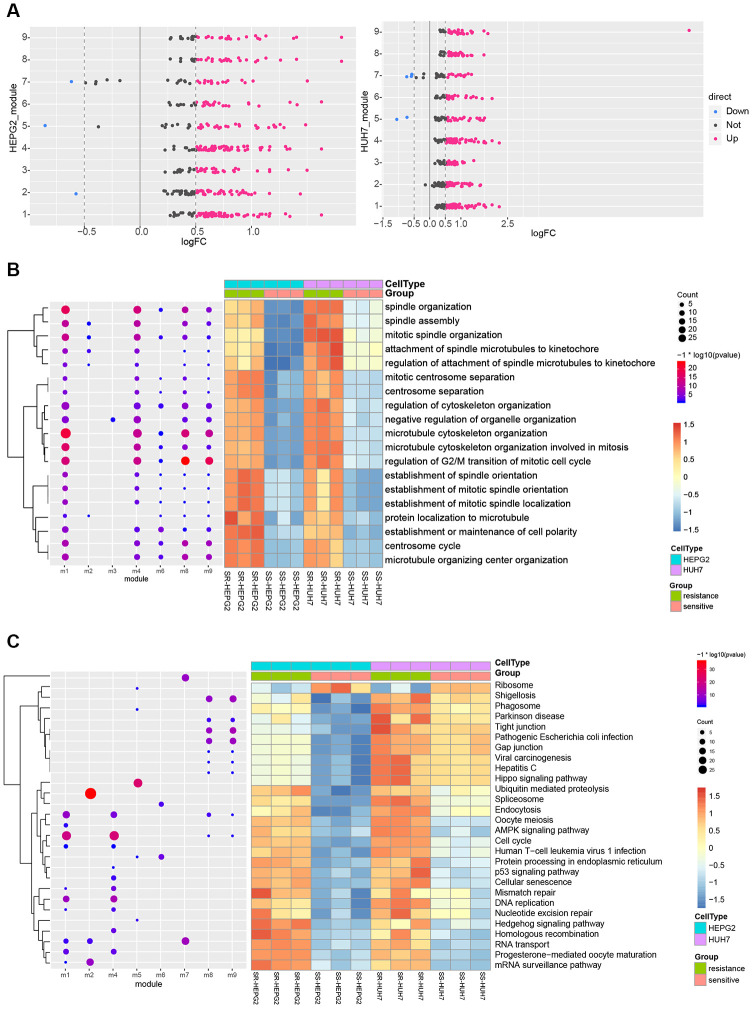
**KEGG pathways and biological processes (BPs) predicted to be perturbed in sorafenib-resistant HCC cells.** (**A**) Modules with similar functions in HepG2 and Huh7 cells. (**B**) Perturbed BPs in HepG2 and Huh7 cells. (**C**) Perturbed KEGG pathways in HepG2 and Huh7 cells.

Nine functional modules were identified in protein-protein interaction (PPI) networks derived from the common DEGs (Supplementary Figure 1, Supplementary Table 1). Nearly all module genes in these two HCC cell lines were up-regulated in SR cells (Figure 3A), suggesting that SR results from dysfunction in multiple modules. There were 2503 biological processes (BPs) (Supplementary Table 2), 560 cellular components (CCs) (Supplementary Table 3), 407 molecular functions (MFs) (Supplementary Table 4) and 57 Kyoto Encyclopedia of Genes and Genomes (KEGG) pathways (Supplementary Table 5). DEGs were involved in various mitosis-related BPs, such as regulation of cytoskeleton organization, microtubule-associated proteins, and the establishment of minimal bundle localization (Figure 3B). The DEGs were involved in various cancer-related pathways as well as virus-associated pathways, such as the cell cycle, Hippo signaling pathway, viral carcinogenesis, and hepatitis C (Figure 3C).

### microRNAs (miRNAs) and transcription factor (TF) may promote the functional modules involved in SR

**Figure 4:**
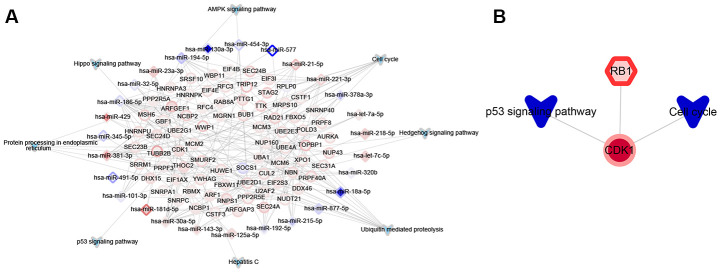
**Significant upstream regulators of functional modules associated with sorafenib resistance in HCC cells.** (**A**) Significant miRNA regulators. The border color of nodes reflects the log (fold change) in HepG2 cells; the inside color reflects the log (fold change) in Huh7 cells. (**B**) Significant transcription factor (TF) regulators. Nodes are colored as in panel A.

The hypergeometric test was used to predict miRNAs and TFs participating in the target functional modules. About 27 miRNAs were identified as potentially regulating functional modules involved in SR (Figure 4A). We did not identify any long non-coding RNAs (lncRNAs) involved in SR. In the network linking TFs with target genes and KEGG pathways, the up-regulated RB1 TF was predicted to promote modules related to SR (Figure 4B). These results suggest that 27 miRNAs and one TF may help mediate SR in HCC.

### Comprehensive landscape of SR in HCC

**Figure 5:**
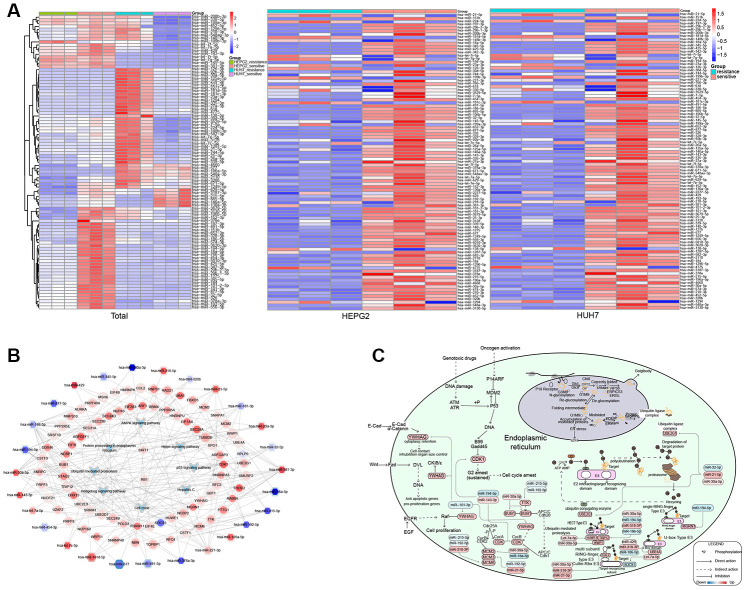
**Potential mechanisms of resistance to sorafenib in hepatocellular carcinoma (HCC).** (**A**) Expression of microRNAs (miRNAs) associated with resistance. (**B**) Comprehensive landscape of miRNAs and transcription factors (TFs) regulating pathways perturbed in sorafenib-resistant HCC. The border color of nodes reflects the log (fold change) in HepG2 cells; the inside color reflects the log (fold change) in Huh7 cells. (**C**) Potential mechanisms of resistance to sorafenib.

A heatmap of miRNAs identified several likely to function in SR in both HCC cell lines (Figure 5A). The comprehensive landscape of miRNAs and one TF promoting KEGG pathways (Figure 5B) identified numerous pathways that may contribute to SR in HCC (Figure 5C). These pathways included the cell cycle, hepatitis C, Hippo signaling pathway, p53 signaling pathway, protein processing in the endoplasmic reticulum, and ubiquitin-mediated proteolysis. In the p53 signaling pathway, we found that CDK1 was up-regulated, which might inhibit cell cycle arrest. We also found that in ubiquitin-mediated proteolysis, miRNAs promoted mainly different types of E3 enzymes, while the expression of UBE2G1 in the E2 enzyme was up-regulated. The E2 and E3 enzymes are involved mainly in substrate transfer. In our study, UBE2G1 was up-regulated in the ubiquitin ligase complex, which might further promote protein processing in the endoplasmic reticulum. YWHAG was also up-regulated, which might inhibit hepatitis C replication within cells, as well as promote anti-apoptotic genes, pro-proliferation genes, cell contact inhibition and organ size control by the Hippo signaling pathway. The cell cycle genes TTK, BUB1, CDK, YWHAG, MCM2, MCM3 and MCM6 were up-regulated, and nearly all were up-regulated by miRNAs, except for SOCS1 in the multi-subunit ring-finger type E3 enzyme of ubiquitin-mediated proteolysis. These miRNAs might promote and activate pathways that contribute to SR in HCC.

## DISCUSSION

Sorafenib remains the only drug approved to treat late-stage HCC, yet its efficacy is limited by primary or acquired resistance. The analyses in the present study identified molecular pathways and networks, as well as their regulators, that may contribute to such resistance. This may guide efforts to reverse resistance, increasing the efficacy of sorafenib.

In the p53 signaling pathway, genetic drugs can inhibit CDK1 and promote the cell cycle. However, in HCC, miR-194-5p and miR-143-3p can mediate high expression of CDK1 and inhibit cell cycle arrest, thus promoting HCC. The increase in MCM2, MCM3, and MCM6 is related to poor performance of the tumor. Removing MCM6 can delay the s/G2 phase of hepatoma cells [9]. Simultaneously, TTK and BUB1 may promote the occurrence of HCC separately [10, 11]. We found that down-regulation of miR-192-5p was associated with SR, consistent with the ability of the miRNA to inhibit HCC [12].

Hepatitis C virus (HCV) is a major cause of HCC [13], and abnormal activation of the Hippo signaling pathway can cause HCC [14]. We found that miR-101-3p negatively regulated YWHAG in the hepatitis C pathway, and that the Hippo signaling pathway promoted HCC. Chaperones in the lumen aid endoplasmic reticulum of protein folding in that compartment. Correctly folded proteins are packaged into transport vesicles, which ferry them to the Golgi complex. In this pathway, miR-32-5p, miR-21-5p and miR-30a-5p up-regulates UBE2G1 in the ubiquitin ligase complex. Thus, abnormalities in the endoplasmic reticulum may contribute to many human diseases [15].

Studies found that SPOP can inhibit the metastasis of HCC cells through the ubiquitin-dependent proteolysis of SENP7 [16]. We identified several SR-associated DEGs related to the E2 (ubiquitin-conjugating) enzymes and E3 (ubiquitin ligase) enzymes. These E2 and E3 DEGs are predicted to be up-regulated by miRNAs, implying that SR involves an increase in ubiquitin-mediated proteolysis. Conversely, the SOCS1 component of the E3 enzyme was down-regulated in SR cells, and lower expression of SOCS1 has been linked to deeper infiltration of HCC cells [17].

We identified several miRNAs that were associated with SR in HCC. Some studies have linked HCC to down-regulation of miR-194-5p [18] and up-regulation of miR-21-5p [19], while miR-192-5p may be clinically useful in diagnosis of the disease [20]. In contrast, miR-221-3p may be unrelated to HCC prognosis [21], whereas miR-429 may be a target in HCC treatment [22], and miR-877-5p may be involved in the pathogenesis of HCC [23]. Relatively few studies have examined miRNAs related to HCC. The present work suggests that miRNAs may promote HCC by altering gene expression, leading to SR in HCC.

Although the present study may provide new insights into SR in HCC, some limitations should be noted. First, the study was based primarily on bioinformatics analysis, so our results should be verified and extended in molecular experiments. Second, we focused on transcriptional regulatory mechanisms of SR, so further work is needed to examine additional mechanisms at the genome level.

## CONCLUSIONS

We identified several functional modules and their potential miRNA and TF that may contribute to SR in HCC. Our results provide numerous leads to guide future mechanistic studies of resistance.

## MATERIALS AND METHODS

### Cell lines

The HCC cell lines HepG2 and Huh7 were obtained (ScienCell, San Diego, California, USA). Three SR and three SS HCC cell lines of HepG2 and Huh7 were obtained based on prior studies [24, 25] SR-HCC cell lines were established through incubation with sorafenib initially at 5 μM, which was increased by 1 μM per week for 1-2 months. SR-HCC cells were maintained in culture in the presence of sorafenib.

### RNA isolation and characterization

Total RNA was isolated from SR- and SS-HCC cell lines using TRIzol^TM^ (Invitrogen, USA) and the RNeasy Mini Kit (Qiagen) according to the manufacturers’ instructions. RNA concentration and purity were assessed using the RNA Nano 6000 Assay Kit on the Agilent 2100 Bioanalyzer (Agilent Technologies, Palo Alto, CA, USA), the NanoDrop system (Thermo Fisher Scientific Inc. Waltham, USA) and 1% agarose gel electrophoresis.

### Strand-specific RNA-seq library preparation and sequencing

Samples (1 μg) with an RNA integrity number ≥ 7 were deep-sequenced using Ribo-Zero™ rRNA removal Kit (Human/Mouse/Rat) /(Yeast) /(Bacteria) (Illumina, San Diego, USA) according to the manufacturer's protocol. Each sample was used to generate a strand-specific RNA-seq library as follows. First the ribosomal RNA in the sample of total RNA was removed, then the RNA was fragmented and reverse-transcribed using ProtoScript II Reverse Transcriptase (New England Biolabs, USA) with random primers and actinomycin D (Solarbio life sciences, USA). The second strand of cDNA was prepared using the Second Strand Synthesis Enzyme Mix with dACG-TP/dUTP (New England Biolabs, USA), then the double-stranded cDNA was purified using the AxyPrep Mag PCR Clean-up (Axygen Biosciences, Inc.), and both ends were repaired using the End Prep Enzyme Mix (NGS Fast DNA Library Prep Set for Illumina, Illumina, San Diego, USA), which added dA-tails. Subsequent T-A ligation created adaptors on both ends.

Adaptor-ligated DNA that contained inserts approximately 300 bp long and that was altogether approximately 360 bp long was isolated, and the second strand (marked with dUTP) was digested using Uracil-Specific Excision Reagent (New England Biolabs, USA). Every sample was amplified by PCR for 11 cycles using primers P5 and P7, which contained sequences that could anneal with the flow cell to perform a bridge PCR. The P7 primer also contained a six-base signature allowing for multiplexing. The PCR products were purified, and library quality was assessed. The resulting libraries were sequenced in 2 × 150 bp paired-end mode on a HiSeq X Ten System (Illumina, San Diego, USA).

### Reading of alignments and transcript assembly

The original sequencing data were subjected to base recognition using the software Bcl2fastq 2.17.1.14 (https://support.illumina.com/content/dam/illuminasupport/documents/documentation/software_documentation/bcl2fastq), yielding “pass filter data”, which were stored in FASTQ file format. Linker and low-quality sequences were deleted from the pass filter data, and the remaining data were compared to the reference genome (GRCH 37) using BWA 0.7.12.

### Sequence alignment and gene expression profiling

Data were cleaned using Cutadapt 1.9.1 and short reads were compared using Hisat2 2.0.1 with default parameters, and the results were stored in SAM file format. Data were reformatted into BAM format using Samtools, and featureCount was used to profile the expression of lncRNAs, miRNAs, and messenger RNAs (mRNAs). The three profiles were normalized using the *limma* package [26] in R 3.5.3.

### Differentially expressed genes (DEGs)/miRNAs/lncRNAs and KEGG pathways associated with SR in HCC

Genes whose expression differences between SR- and SS-HCC cell lines were associated with P < 0.05 after adjustment by the false discovery rate (FDR) were considered DEGs. Up- and down-regulated DEGs common to SR-HepG2 and SR-Huh7 lines were subjected to further analyses. Targets of sorafenib in the DrugBank database (https://www.drugbank.ca/) [27] were obtained. We also identified differentially expressed miRNAs and lncRNAs in SR-HepG2 and SR-Huh7 compared to SS-HepG2 and SS-Huh7 cell lines, respectively. Normalized mRNA expression profiles were subjected to gene set enrichment analysis using GSEA software [11] in JAVA. The reference gene set c2.cp.kegg.v6.2. symbols.gmt was taken from the Molecular Signatures Database (MSigDB) [12]. A nominal value of P < 0.05 was considered to be statistically significant.

### Analysis of PPI networks and modules related to SR in HCC

PPI networks with a combined score > 500 based on shared DEGs were extracted from the STRING database (https://string-db.org/) [28]. Modules were extracted from the PPI network using the ClusterONE [29] plug-in in Cytoscape [30], with a minimum cytoscape set of 30. The *ClusterProfiler* package [31] in R was applied to explore potential BPs, CCs, MFs and KEGG pathways enriched in the modules. BPs and pathways associated with an FDR-adjusted P < 0.05 were considered significant.

### Exploring upstream regulators of the functional modules and transcription regulatory network associated with SR in HCC

Interactions between lncRNAs or miRNAs and their target genes were obtained from the RNAInter database [32], and interactions between TFs and their target genes were obtained from the TRRUST v2 database [33]. Interactions between regulators and target functional modules were identified using the hypergeometric test in R, and interactions showing a quantity >2 and P < 0.05 were considered significant. All these results were used to construct a transcription regulatory network associated with SR in HCC.

## Supplementary Material

Supplementary Figure 1

Supplementary Table 1

Supplementary Table 2

Supplementary Table 3

Supplementary Table 4

Supplementary Table 5
