# Supplementary material for: Exploring the mechanism of resistance to sorafenib in two hepatocellular carcinoma cell lines
Source: Aging (Albany NY). 2020 Nov 21;12(23):24255–69. doi: 10.18632/aging.104195 (PMC7762478; doi:10.18632/aging.104195)
Supplement: Supplementary Figure 1 [file aging-12-104195-s001.pdf]

SUPPLEMENTARY FIGURE

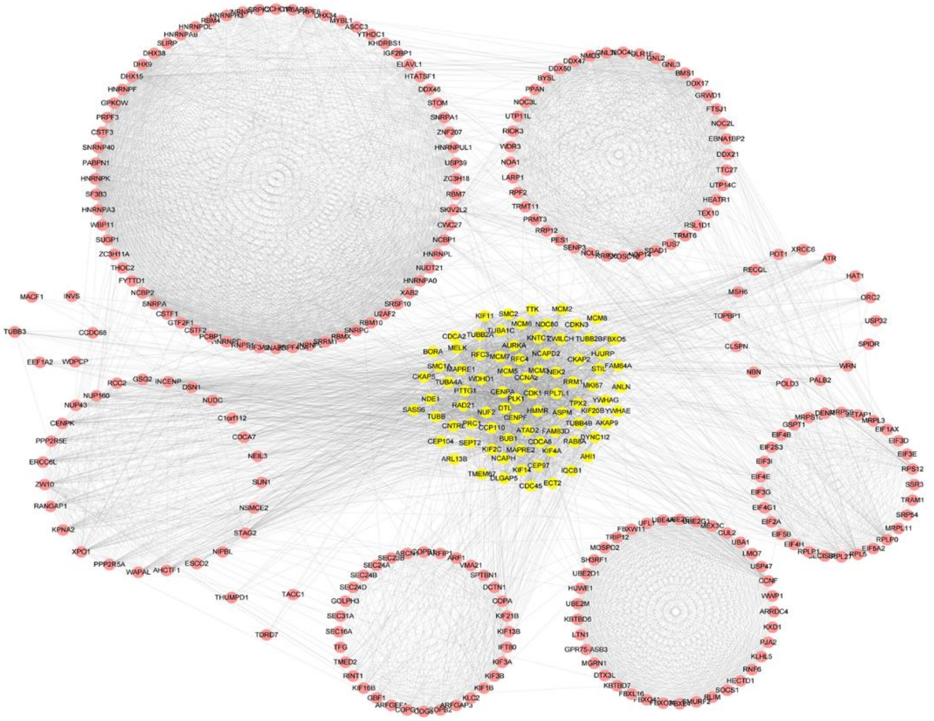

Supplementary Figure 1. Modular analysis of a protein-protein interation network. Yellow nodes indicated genes common to at least two modules.
