# Supplementary material for: Exploring the mechanism of resistance to sorafenib in two hepatocellular carcinoma cell lines
Source: Aging (Albany NY). 2020 Nov 21;12(23):24255–69. doi: 10.18632/aging.104195 (PMC7762478; doi:10.18632/aging.104195)
Supplement: Supplementary Table 1 [file aging-12-104195-s002.pdf]

SUPPLEMENTARY TABLE

Supplementary Table 1. Functional modules, identified using ClusterONE, in the protein-protein interaction network of differentially expressed genes common to HepG2 and Huh7 lines.

Cluster	Size	Members
1	84	XPO1, ZWILCH, RFC3, RFC4, PLK1, NUP160, SMC1A, RAD21, STAG2, SUN1, MAPRE1, PPP2R5A, ESCO2, WDHD1, NIPBL, WAPAL, KNTC1, INCENP, ZW10, RRM1, TTK, KPNA2, PTTG1, NUP43, RANGAP1, TPX2, DTL, PPP2R5E, STIL, SMC2, MCM3, MCM5, MCM2, MCM7, MCM6, MCM8, CDKN3, CDK1, NCAPD2, GSG2, RCC2, KIF2C, CCNA2, NCAPH, NUDC, PRC1, NSMCE2, NUF2, NDC80, MELK, KIF11, CDC45, KIF4A, CDCA8, CENPA, DLGAP5, HJURP, ECT2, CDCA7, FBXO5, C1orf112, CENPF, HMMR, CDCA2, MKI67, NEK2, NDE1, KIF20B, KIF14, FAM64A, CENPK, NEIL3, FAM83D, ERCC6L, DSN1, CKAP2, ASPM, BORA, BUB1, AURKA, ATAD2, ANLN, CKAP5, AHCTF1
2	68	SRRM1, MYBL1, U2AF2, ZC3H11A, USP39, PRPF3, SNRPA, HTATSF1, SKIV2L2, RBM7, NCBP2, NCBP1, PRPF8, NUDT21, SNRPC, SNRPA1, HNRNPDL, XAB2, SF3B3, HNRNPL, HNRNPA0, SUGP1, SRSF10, RBM10, SF3A2, RBM4, GTF2F1, CWC27, PABPN1, DDX46, DHX9, RNPS1, DHX38, DNAJC8, HNRNPA3, PRPF40A, SNRNP40, GPKOW, PCBP1, HNRNPU, WBP11, HNRNPUL1, ELAVL1, HNRNPR, RBMX, HNRNPH2, HNRNPF, HNRNPK, DHX15, ZC3H18, HNRNPH3, HNRNPAB, STOM, YTHDC1, THOC2, IGF2BP1, ZNF207, SRPK2, KHDRBS1, FYTTD1, CSTF2, CSTF3, CSTF1, ZCCHC10, SLIRP, DHX34, ASCC3, ATP6AP2
3	42	TEX10, RPL7L1, RRP12, SENP3, PPAN, PES1, RPF2, SDAD1, NMD3, PRMT3, TTC27, NOP14, NOL9, WDR3, NOC4L, NOC3L, LARP1, UTP14C, TRMT11, EXOSC10, HEATR1, NOC2L, UTP11L, RIOK3, POLR1E, RSL1D1, NOA1, TRMT6, RRP36, PUS7, BYSL, DDX50, GNL3, FTSJ1, DDX21, EBNA1BP2, GNL2, DDX47, GRWD1, GNL3L, DDX17, BMS1
4	70	ZWILCH, RFC3, SPIDR, WRN, PALB2, POLD3, RFC4, PLK1, RECQL, POT1, SMC1A, RAD21, XRCC6, NBN, WDHD1, KNTC1, RRM1, TTK, PTTG1, TOPBP1, TPX2, HAT1, DTL, USP32, STIL, SMC2, MCM3, MCM5, MCM2, MCM7, ORC2, MCM6, MCM8, CDKN3, CDK1, NCAPD2, KIF2C, CCNA2, NCAPH, PRC1, NUF2, NDC80, MELK, MSH6, KIF11, CDC45, KIF4A, CDCA8, CENPA, DLGAP5, HJURP, CLSPN, ECT2, FBXO5, CENPF, HMMR, CDCA2, MKI67, NEK2, KIF20B, KIF14, ATR, FAM64A, FAM83D, CKAP2, ASPM, BUB1, AURKA, ATAD2, ANLN
5	37	UBE2D1, WWP1, SOCS1, UBE4A, KXD1, PJA2, RLIM, RNF6, MOSPD2, TRIP12, SMURF2, FBXO36, FBXO41, UBA1, USP47, UBE2M, LMO7, MEX3C, KBTBD7, UBE2E3, KLHL5, UFL1, LTN1, SH3RF1, MGRN1, UBE2G1, HECTD1, HUWE1, KBTBD6, FBXL4, FBXW11, GPR75-ASB3, FBXL16, DTX3L, CCNF, CUL2, ARRDC4
6	33	TMED2, SPTBN1, IFT80, KIF3A, KIF3B, TFG, VMA21, SEC24A, SEC31A, SEC24D, SEC24B, SEC16A, KIF21B, RINT1, DCTN1, GOLPH3, COPG1, KIF1B, ARFIP1, COPB1, COPA, ARFGEF1, GBF1, KLC2, KIF20B, ARFGAP3, COPB2, KIF13B, KIF16B, COG5, SEC23B, ARCN1, ARF1
7	30	MRPS9, MRPS10, MRPL11, RPLP0, SRP54, RPL5, RPL27, RPL7L1, RPS12, RPLP1, MRPL3, SSR3, TRAM1, EIF5A2, EIF2A, EIF5B, EIF4G1, EIF2S3, EIF4E, EIF3G, EIF3E, EIF3I, EIF3D, SECISBP2, EIF4H, EIF4B, METAP1, GSPT1, DENR, EIF1AX
8	33	TACC1, SEPT2, MAPRE2, MAPRE1, RAB8A, YWHAE, TPX2, YWHAG, IQCB1, THUMPD1, CEP104, CEP97, SASS6, TUBB4B, TUBB, TUBB2A, TUBB2B, DYNC1I2, TUBA4A, TUBA1C, TDRD7, CCP110, HMMR, NEK2, NDE1, BORA, AURKA, CNTRL, CKAP5, AKAP9, TMEM67, ARL13B, AHI1
9	33	SEPT2, MAPRE2, MACF1, MAPRE1, RAB8A, YWHAE, TUBB3, YWHAG, IQCB1, CEP104, CEP97, SASS6, TUBB4B, TUBB, TUBB2A, TUBB2B, INVS, DYNC1I2, TUBA4A, TUBA1C, CCP110, NEK2, NDE1, EEF1A2, BORA, CNTRL, CKAP5, AKAP9, TMEM67, CCDC68, WDPCP, ARL13B, AHI1
