# Supplementary material for: Exploring the mechanism of resistance to sorafenib in two hepatocellular carcinoma cell lines
Source: Aging (Albany NY). 2020 Nov 21;12(23):24255–69. doi: 10.18632/aging.104195 (PMC7762478; doi:10.18632/aging.104195)
Supplement: Supplementary Table 5 [file aging-12-104195-s006.pdf]

SUPPLEMENTARY TABLE

Supplementary Table 5. The Kyoto Encyclopedia of Genes and Genomes pathways enriched in sorafenib-resistant samples.

module	ID	Description	p.adjust	geneID	Count
modulem1	hsa04152	AMPK signaling pathway	0.041950813	PPP2R5A/PPP2R5E/CCNA2	3
modulem1	hsa04110	Cell cycle	7.73E-19	STAG2/MCM2/MCM3/MCM5/MCM6/MCM7/PLK1/RAD21/BUB1/TTK/SMC1A/CDC45/CCNA2/PTTG1/CDK1	15
modulem1	hsa03030	DNA replication	5.10E-10	MCM2/MCM3/MCM5/MCM6/MCM7/RFC3/RFC4	7
modulem1	hsa05166	Human T-cell leukemia virus 1 infection	0.141651764	XPO1/CCNA2/PTTG1	3
modulem1	hsa03430	Mismatch repair	0.016348116	RFC3/RFC4	2
modulem1	hsa03015	mRNA surveillance pathway	0.141651764	PPP2R5A/PPP2R5E	2
modulem1	hsa03420	Nucleotide excision repair	0.049602581	RFC3/RFC4	2
modulem1	hsa04114	Oocyte meiosis	4.74E-09	FBXO5/PLK1/PPP2R5A/PPP2R5E/AURKA/BUB1/SMC1A/PTTG1/CDK1	9
modulem1	hsa04914	Progesterone-mediated oocyte maturation	0.000206509	PLK1/AURKA/BUB1/CCNA2/CDK1	5
modulem1	hsa03013	RNA transport	0.016348116	NUP160/NUP43/RANGAP1/XPO1	4
modulem2	hsa03015	mRNA surveillance pathway	1.30E-09	SRRM1/RNPS1/NUDT21/CSTF1/CSTF2/CSTF3/NCBP2/NCBP1/PABPN1	9
modulem2	hsa03013	RNA transport	0.003546577	SRRM1/RNPS1/NCBP2/NCBP1/THOC2	5
modulem2	hsa03040	Spliceosome	1.00E-35	PRPF8/USP39/SRSF10/U2AF2/DHX15/HNRNPA3/NCBP2/SF3B3/RBMX/HNRNPK/HNRNPU/NCBP1/PCBP1/WBP11/PRPF40A/XAB2/THOC2/SNRPA/SNRPA1/SNRPC/SF3A2/PRPF3/SNRNP40/DHX38/DDX46	25
modulem3	hsa03008	Ribosome biogenesis in eukaryotes	1.35E-13	WDR3/GNL3/GNL2/NMD3/GNL3L/HEATR1/UTP14C/BMS1	8
modulem3	hsa03020	RNA polymerase	0.077470859	POLR1E	1
modulem4	hsa04110	Cell cycle	8.91E-21	MCM2/MCM3/MCM5/MCM6/MCM7/ORC2/PLK1/ATR/RAD21/BUB1/TTK/SMC1A/CDC45/CCNA2/PTTG1/CDK1	16
modulem4	hsa04218	Cellular senescence	0.009542975	NBN/ATR/CCNA2/CDK1	4
modulem4	hsa03030	DNA replication	4.86E-12	POLD3/MCM2/MCM3/MCM5/MCM6/MCM7/RFC3/RFC4	8
modulem4	hsa03460	Fanconi anemia pathway	0.053740596	ATR/PALB2	2
modulem4	hsa03440	Homologous recombination	7.96E-05	POLD3/TOPBP1/NBN/PALB2	4
modulem4	hsa05166	Human T-cell leukemia virus 1 infection	0.113368193	ATR/CCNA2/PTTG1	3
modulem4	hsa03430	Mismatch repair	9.11E-06	POLD3/MSH6/RFC3/RFC4	4
modulem4	hsa03450	Non-homologous end-joining	0.113368193	XRCC6	1
modulem4	hsa03420	Nucleotide excision repair	0.002713314	POLD3/RFC3/RFC4	3
modulem4	hsa04114	Oocyte meiosis	2.87E-06	FBXO5/PLK1/AURKA/BUB1/SMC1A/PTTG1/CDK1	7
modulem4	hsa04115	p53 signaling pathway	0.082941236	ATR/CDK1	2
modulem4	hsa04914	Progesterone-mediated oocyte maturation	0.00012879	PLK1/AURKA/BUB1/CCNA2/CDK1	5
modulem5	hsa04340	Hedgehog signaling pathway	0.047695487	FBXW11/SMURF2	2
modulem5	hsa05012	Parkinson disease	0.197409651	UBA1/UBE2G1	2
modulem5	hsa04141	Protein processing in endoplasmic reticulum	0.207742282	UBE2D1/UBE2G1	2
modulem5	hsa05131	Shigellosis	0.065360875	FBXW11/UBE2D1	2
modulem5	hsa04120	Ubiquitin mediated proteolysis	3.36E-22	HUWE1/UBE2E3/WWP1/FBXW11/MGRN1/SMURF2/UBA1/UBE2D1/UBE2G1/CUL2/SOCS1/UBE2M/TRIP12/UBE4A	14
modulem6	hsa04144	Endocytosis	0.002587885	ARFGEF1/ARFGAP3/ARF1/GBF1	4
modulem6	hsa04141	Protein processing in endoplasmic reticulum	4.29E-05	SEC24B/SEC23B/SEC24A/SEC31A/SEC24D	5

modulem7	hsa03010	Ribosome	1.43E-09	MRPL3/MRPS10/RPL5/RPL27/RPLP0/RPLP1/RPS12/MRPS9/MRPL11	9
modulem7	hsa03013	RNA transport	1.59E-10	EIF1AX/EIF2S3/EIF4B/EIF4E/EIF4G1/EIF3E/EIF3D/EIF3G/EIF3I/EIF5B	10
modulem8	hsa04210	Apoptosis	0.047212144	TUBA4A/TUBA1C	2
modulem8	hsa04110	Cell cycle	0.046453039	YWHAE/YWHAG	2
modulem8	hsa04540	Gap junction	9.14E-09	TUBB4B/TUBB/TUBB2B/TUBA4A/TUBB2A/TUBA1C	6
modulem8	hsa05160	Hepatitis C	0.047212144	YWHAE/YWHAG	2
modulem8	hsa04390	Hippo signaling pathway	0.047212144	YWHAE/YWHAG	2
modulem8	hsa04114	Oocyte meiosis	0.003949079	AURKA/YWHAE/YWHAG	3
modulem8	hsa05130	Pathogenic Escherichia coli infection	1.50E-09	TUBB4B/TUBB/TUBB2B/TUBA4A/TUBB2A/TUBA1C	6
modulem8	hsa04145	Phagosome	6.08E-09	TUBB4B/DYNC1I2/TUBB/TUBB2B/TUBA4A/TUBB2A/TUBA1C	7
modulem8	hsa04530	Tight junction	0.007179351	RAB8A/TUBA4A/TUBA1C	3
modulem8	hsa05203	Viral carcinogenesis	0.068829321	YWHAE/YWHAG	2
modulem9	hsa04210	Apoptosis	0.057983071	TUBA4A/TUBA1C	2
modulem9	hsa04110	Cell cycle	0.057983071	YWHAE/YWHAG	2
modulem9	hsa04540	Gap junction	1.86E-10	TUBB3/TUBB4B/TUBB/TUBB2B/TUBA4A/TUBB2A/TUBA1C	7
modulem9	hsa05160	Hepatitis C	0.057983071	YWHAE/YWHAG	2
modulem9	hsa04390	Hippo signaling pathway	0.057983071	YWHAE/YWHAG	2
modulem9	hsa04114	Oocyte meiosis	0.057983071	YWHAE/YWHAG	2
modulem9	hsa05130	Pathogenic Escherichia coli infection	1.83E-11	TUBB3/TUBB4B/TUBB/TUBB2B/TUBA4A/TUBB2A/TUBA1C	7
modulem9	hsa04145	Phagosome	1.86E-10	TUBB3/TUBB4B/DYNC1I2/TUBB/TUBB2B/TUBA4A/TUBB2A/TUBA1C	8
modulem9	hsa04530	Tight junction	0.012086629	RAB8A/TUBA4A/TUBA1C	3
modulem9	hsa05203	Viral carcinogenesis	0.084210805	YWHAE/YWHAG	2
